# The Clinical Utility of a Precision Medicine Blood Test Incorporating Age, Sex, and Gene Expression for Evaluating Women with Stable Symptoms Suggestive of Obstructive Coronary Artery Disease: Analysis from the PRESET Registry

**DOI:** 10.1089/jwh.2018.7203

**Published:** 2019-05-17

**Authors:** Burcu Gul, Alexandra Lansky, Matthew J. Budoff, David Sharp, Bruce Maniet, Lee Herman, Jane Z. Kuo, Lin Huang, Mark Monane, Joseph A. Ladapo

**Affiliations:** ^1^ Section of Cardiology, Yale University, New Haven, Connecticut.; ^2^ Division of Cardiology, UCLA, Torrance, California.; ^3^ Doctors for Health, Omaha, Nebraska.; ^4^ Bells Medical Clinic, Bells, Texas.; ^5^ Johns Creek Primary Care, Suwanee, Georgia.; ^6^ CardioDx, Inc., Redwood City, California.; ^7^ Division of General Internal Medicine and Health Services Research, David Geffen School of Medicine, UCLA, Los Angeles, California.

**Keywords:** coronary artery disease, women, diagnosis, age/sex/gene expression score, registry

## Abstract

***Background:*** Evaluating women with symptoms suggestive of coronary artery disease (CAD) remains challenging. A blood-based precision medicine test yielding an age/sex/gene expression score (ASGES) has shown clinical validity in the diagnosis of obstructive CAD. We assessed the effect of the ASGES on the management of women with suspected obstructive CAD in a community-based registry.

***Materials and Methods:*** The prospective PRESET (A Registry to Evaluate Patterns of Care Associated with the Use of Corus^®^ CAD in Real World Clinical Care Settings) Registry (NCT01677156) enrolled 566 patients presenting with symptoms suggestive of stable obstructive CAD from 21 United States primary care practices from 2012 to 2014. Demographics, clinical characteristics, and referrals to cardiology or further functional and/or anatomical cardiac studies after ASGES testing were collected for this subgroup analysis of women from the PRESET Registry. Patients were followed for 1-year post-ASGES testing.

***Results:*** This study cohort included 288 women with a median age 57 years. The median body mass index was 29.2, with hyperlipidemia and hypertension present in 48% and 43% of patients, respectively. Median ASGES was 8.5 (range 1–40), with 218 (76%) patients having low (≤15) ASGES. Clinicians referred 9% (20/218) low ASGES versus 44% (31/70) elevated ASGES women for further cardiac evaluation (odds ratio 0.14, *p* < 0.0001, adjusted for patient demographics and clinical covariates). Across the score range, higher ASGES were associated with a higher likelihood of posttest cardiac referral. At 1-year follow-up, low ASGES women experienced fewer major adverse cardiac events than elevated ASGES women (1.3% vs. 4.2% respectively, *p* = 0.16).

***Conclusions:*** Incorporation of ASGES into the diagnostic workup demonstrated clinical utility by helping clinicians identify women less likely to benefit from further cardiac evaluation.

## Introduction

Sex-specific differences in cardiovascular disease contribute to unique diagnostic challenges in the evaluation of women with suspected coronary artery disease (CAD). Women with suspected CAD are more likely than men to present with atypical symptoms, and noninvasive diagnostic testing—recommended by the American Heart Association (AHA)/American College of Cardiology (ACC)—is more likely to yield a falsely negative result in women compared with men.^1^ The workup for suspected CAD in women is also associated with errors of commission—in the form of unnecessary testing—as well as omission—with undertesting in appropriate patients—both of which contribute to suboptimal care.^2–5^

Current AHA/ACC guidelines recommend noninvasive exercise electrocardiogram (ECG) with or without imaging for diagnostic evaluation. However, advancements in precision medicine hold significant promise for improving care and health outcomes among women presenting with typical and atypical angina symptoms.^6, 7^ A blood-based age/sex/gene expression score (ASGES) for the evaluation of obstructive CAD incorporates several key features of precision medicine—including integration of age- and sex-specific patient characteristics as well as molecular genomics and compatibility with web portal/electronic health records—thereby providing physicians with additional data to assess patients with symptoms suggestive of obstructive CAD. The ASGES test, ranging from a score of 1–40, with higher scores associated with an increased current likelihood of obstructive CAD, has been validated in multiple studies involving nondiabetic patients referred for invasive coronary angiography (ICA) and myocardial perfusion imaging (MPI) and has been shown to have a 96% negative predictive value in a combined cohort of men and women in the COMPASS (Coronary Obstruction Detection by Molecular Personalized Gene Expression) study.^8–10^

When compared to MPI, the ASGES independently improved the diagnosis evaluation of obstructive CAD in both men and women, whereas MPI was a less reliable test in women, with rates of angiographically proven obstructive CAD being similar among women with positive and negative MPI (22% vs. 18.5%).^8^ Furthermore, in the recent National Heart, Lung, and Blood Institute-sponsored PROMISE (Prospective Multicenter Imaging Study for Evaluation of Chest Pain) substudy, the ASGES test was performed in 2,380 nondiabetic patients presenting with symptoms suggestive of obstructive CAD. In this study, higher ASGES scores were associated with higher current likelihood of obstructive CAD as well as higher likelihood of the composite endpoint of death, myocardial infarction (MI), unstable angina, or revascularization procedures at 2-year follow-up.^11^ Furthermore, a recent 2017 AHA Scientific Statement on the Expressed Genome highlighted the ASGES and its clinical value in the evaluation of patients with suspected obstructive CAD.^12^

The ASGES test is the only sex-specific diagnostic tests designed to risk stratify patients with symptoms suggestive of obstructive CAD. In this study, we examined all women in the community-based PRESET (A Registry to Evaluate Patterns of Care Associated with the Use of Corus^®^ CAD in Real World Clinical Care Settings; PRESET Registry, NCT01677156) Registry to determine the clinical utility of the ASGES, based on its potential effects on medical decision-making and referrals to cardiology or advanced cardiac testing.^13^ The PRESET Registry differs from the previously published pooled cohort of women from REGISTRY I and IMPACT studies.^14^ The IMPACT-PCP (Investigation of a Molecular Personalized Coronary Gene Expression Test on Primary Care Practice Pattern) study incorporated a rigorous clinical trial design, including more restrictive entry criteria and predefined follow-up time points. In contrast, PRESET used a registry-type design to measure clinical effectiveness and more accurately reflects real-world decision-making and outcomes. For these same reasons, it is possible that the results in this PRESET subgroup are more generalizable.

## Materials and Methods

### Study tools

The age/sex/gene expression blood test (Corus CAD^®^, CardioDx, Inc., Redwood City, CA) is intended for use in stable patients with a history of chest pain or suspected anginal equivalents. The ASGES test is not intended for use in patients with diabetes, systemic infectious or systemic inflammatory conditions, or who are currently taking steroids, immunosuppressive agents, or chemotherapeutic agents.^15^

The ASGES test is a commercially available, quantitative test measuring expression levels of 23 genes from a peripheral blood sample. These genes are selectively expressed in multiple types of circulating cells, including neutrophils, natural killer cells, as well as B and T lymphocytes. These cells play supporting roles in both adaptive and innate immune responses in atherosclerosis.^16^ Whole blood samples were collected in PAXgene Blood RNA Tube (PreAnalytiX, Hombrechtikon, Switzerland) and were processed as previously described.^15^

Previously validated sex-specific algorithms with age and gene expression inputs are used to generate the ASGES, ranging in value from 1 to 40.^15^ Each value is associated with the current likelihood of obstructive CAD: higher ASGES are associated with higher current likelihood of obstructive CAD, which is defined as at least one atherosclerotic plaque causing ≥50% luminal diameter stenosis in a major coronary artery (≥1.5 mm lumen diameter), and has been correlated with invasive quantitative coronary angiography or core laboratory coronary computed tomography angiography (CCTA) (≥2.0 mm).^17^ Patients in the ASGES validation studies were stratified into low ASGES (≤15) (estimated probability of obstructive CAD ≤8%) and elevated ASGES (>15) (estimated probability of obstructive CAD >8%) subgroups for further analysis. A low ASGES score (≤15) was found to have a 96% negative predictive value in a combined population of men and women (*N* = 431) for determining a patient's current likelihood of having obstructive CAD and is associated with low rates (0.5%) of major adverse cardiac event (MACE) or revascularization during 6 month follow-up in the COMPASS study.^10^

### Study design

The prospective PRESET Registry enrolled stable, nondiabetic adult patients without a history of CAD from 21 United States primary care practices from August 2012 to August 2014. Quorum Review, Inc. granted Institutional Review Board (IRB) approval, and all enrolled patients signed an IRB-approved informed consent form. Clinicians and their office staffs at each of the primary care sites were educated and trained on the use and interpretation of the ASGES through a standardized, in-service program. Clinicians solely determined whether their patients met the intended use criterion and received ASGES testing.

The study group comprised symptomatic patients who presented to primary care clinicians for the evaluation of suspected obstructive CAD and underwent ASGES testing. Patients were classified as having typical angina symptoms, such as substernal chest discomfort, aggravation with exertion, and alleviation with rest as well as dyspnea, or atypical angina symptoms, such as, palpitations, malaise, and fatigue. At baseline, data regarding patient demographics and medical history were recorded. On follow-up, ASGES results, referrals to cardiology or advanced cardiac testing, and major adverse cardiac outcomes were measured. A total of 566 patients were enrolled in the PRESET Registry: this predefined analysis focused on the 288 (51%) subgroup of women patients.

### Study outcomes

The primary objective of the study was to evaluate the ASGES score and its effects on medical decision-making—specifically, referrals to cardiology or further cardiac testing—in a community-based cardiovascular patient registry at 45 day follow-up. Further cardiac testing was defined as exercise treadmill testing (ETT), exercise stress echocardiogram (ECHO), MPI, CCTA, or ICA. Follow-up of registry patients was conducted at 1-year by chart review to assess the incidence of MACE, defined as a composite of stroke/transient ischemic attack (TIA), MI, and cardiac-related death.

### Statistical analyses

Descriptive statistics for univariate analyses, including means and standard deviations, counts and percentages, and counts of missing data records, were calculated for continuous and categorical variables, as appropriate. Tests for statistical association between cardiac referral and ASGES classification as a binary variable (predefined as low score [<15] and elevated score [>15]) as well as a continuous variable from 1 to 40 were performed using logistic regression, with and without adjustment for participant characteristics and clinical covariates (smoking, race/ethnicity, body mass index [BMI], hypertension, and hyperlipidemia). Odds ratios (ORs), 95% confidence intervals, and *p*-values were used to assess statistical significance of the results. All analyses were performed using R (version 3.0.2).^18^

## Results

The cohort of 288 women had a median age of 57 years (interquartile range 44–68) and was predominantly white (81%). The median BMI was 29.2, 34% were recent smokers, and hyperlipidemia and hypertension were present in 48% and 43% of patients, respectively. Approximately one-third (35%) had typical angina symptoms at the time of presentation. The median ASGES was 8.5 (range, 1–40) (Table 1).

**Table 1.** Clinical and Demographic Characteristics of Women in the PRESET Registry (*N* = 288)

Age	Median 57 (25–96)
Race	
White	234 (81%)
Black	44 (15%)
Asian	5 (2%)
American Indian or Alaska Native	1 (0%)
Other	4 (1%)
BMI (*n* = 285)	Median 29.2 (15.3–67.29)
Systolic BP (*n* = 286)	Mean 128.5 (±16.86)
Diastolic BP (*n* = 286)	Mean 75.67 (±11.49)
Smoker status	
Current	47 (16%)
Quit within last month	1 (0%)
Quit more than 1 month ago	51 (18%)
Never	189 (66%)
Anginal symptoms	
Typical	102 (35%)
Atypical	186 (65%)
Medical history	
Hypertension	124 (43%)
Hyperlipidemia	138 (48%)
Carotid artery disease	5 (2%)
Peripheral artery disease	1 (0%)
Liver disease	1 (0%)
Cancer	10 (3%)
Postmenopausal	184 (64%)
Systemic inflammatory	2 (1%)
Arrhythmia	8 (3%)
Respiratory conditions	31 (11%)
Autoimmune or Inflammatory	8 (3%)
ASGES	median 8.5 (range 1–40)

PRESET, A Registry to Evaluate Patterns of Care Associated with the Use of Corus^®^ CAD in Real-World Clinical Care Settings; BMI, body mass index; BP, blood pressure; ASGES, age/sex/gene expression score.

**FIG. 1.**
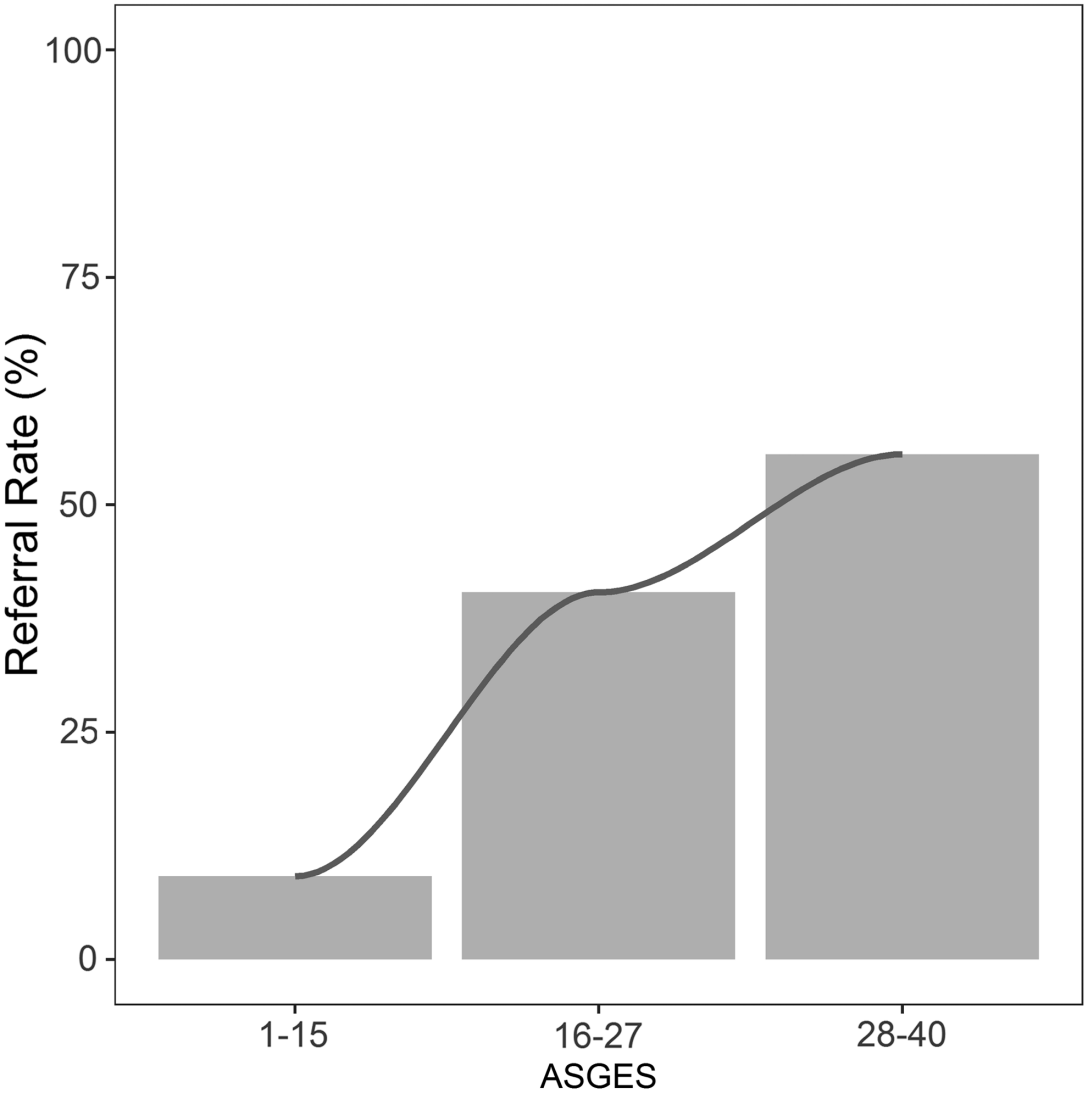
Relationship between ASGES group and percentage of referral to cardiology or advanced cardiac testing. ASGES, age/sex/gene expression score.

After ASGES testing, 218 patients (76%) had low scores. Clinicians referred 20/218 (9%) patients with low scores versus 31/70 (44%) patients with elevated scores to cardiology or advanced cardiac testing (ETT, ECHO, MPI, CCTA, and ICA) (OR 0.13, *p* ≤ 0.0001 on univariate analysis) (Table 2). In multivariate analyses adjusting for clinical covariates BMI, smoking status, hypertension, and hyperlipidemia, the association between low ASGES and low rate of referral persisted (OR 0.14, *p* < 0.0001), while other demographic factors were not associated with cardiac referral (Table 3). Analysis of the ASGES as a continuous variable showed that the rate of cardiac referral increased proportionally with the score, with referral rates of 9% (20/218), 40% (21/52), and 55% (10/18) among low- (ASGES 1–15), intermediate- (ASGES 16–27), and high-score (ASGES 28–40) patients, respectively (*p* < 0.0001) (Fig. 1). For every five-point increase in ASGES, the adjusted OR of referral increased by 1.54-fold in the multivariate model (*p* < 0.0001).

**Table 2.** Referral to Cardiology or Advanced Cardiovascular Testing (ETT, ECHO, MPI, CCTA, and ICA) (Univariate Analysis)

	*Referred to cardiologist or further cardiac testing*	*Not referred to cardiologist or further cardiac testing*	*OR (95% CI)*	p*-Value*
ASGES ≤15 (*n* = 218)	20 (9%)	198 (91%)	0.13 (0.06–0.26)	<0.0001
ASGES >15 (*n* = 70)	31 (44%)	39 (56%)		

ETT, exercise treadmill testing; ECHO, exercise stress echocardiogram; MPI, myocardial perfusion imaging; CCTA, coronary computed tomography angiogram; ICA, invasive coronary angiography; CI, confidence interval; OR, odds ratio.

**Table 3.** Referral to Cardiology or Advanced Cardiovascular Testing (ETT, ECHO, MPI, CCTA, and ICA) (Multivariate Analysis)

*Characteristic*	*OR*	*95% CI*		p*-Value*
Low ASGES score (≤15)	0.14	0.06	0.29	<0.0001
Current smoker	0.69	0.22	1.85	0.495
Hypertension	1.37	0.66	2.87	0.396
Dyslipidemia	1.09	0.51	2.32	0.816
White race	0.57	0.24	1.41	0.207
BMI ≥30	1.64	0.82	3.36	0.163

There were six patients (6/288, 2.1%) with MACE within the 1-year follow-up period. There were four women (4/288, 1.4%), who experienced a stroke/TIA, and two women (2/288, 0.7%), who died. Major adverse cardiac outcomes occurred in three patients (3/218, 1.3%) with low ASGES and three patients (3/70, 4.2%) with elevated ASGES (*p* = 0.16).

## Discussion

A major challenge faced by primary care providers is the evaluation of typical and atypical chest pain in women, because conventional diagnostic approaches are limited in their ability to rule-out or rule-in CAD in the outpatient setting. In this prospective, community-based registry study of nondiabetic women evaluated for suspected stable CAD, the addition of the ASGES test, derived from a sex-specific algorithm with age and gene expression inputs, demonstrated clinical utility through its significant association with clinical decision-making. Women with low ASGES had an 86% decreased odds of referral for further cardiac evaluation compared with women with elevated ASGES. In addition, proportionally higher rates of referral among higher ASGES patients were noted across the ASGES range of 1–40. One-year follow-up data in this cohort support the safety of this diagnostic approach among patients with suspected obstructive CAD. Thus, an ASGES-guided strategy may reduce unnecessary cardiac stress testing and CCTA among women.

The purpose of the ASGES is not to supplant a patient's history, but rather to aid in the diagnostic evaluation, similar to the manner that functional/anatomical cardiac testing is used. Based on Diamond–Forrester estimates and the prevalence of symptoms in PRESET Registry, most women in this study likely had intermediate pretest probability of CAD, which corresponds to a probability of CAD of 10%–90%, according to AHA guidelines.^19,20^ We did not estimate Framingham risk scores because the ASGES informs the current likelihood of obstructive CAD, in contrast to Framingham scores, which provide information about prognosis.

Current AHA guidelines recommend that patients with intermediate pretest probability of CAD be referred for cardiac stress testing or CCTA, which routinely requires patients to take substantial time away from work or other activities, and frequently also requires radiation and contrast exposure.^21,22^ However, ∼75% of the patients in this study had low ASGES, classifying them as patients with low current likelihood of obstructive CAD as well as a low 1.3% MACE rate at 1-year follow-up. One-year MACE rate in women with low ASGES in this study was similar to the annual mortality rate after a negative MPI or CCTA as noted in the PROMISE substudy. In addition, the PROMISE substudy demonstrated that patients with an ASGES ≤15 had a composite MACE rate similar to those with negative noninvasive test results during 2-year follow-up (3.2% vs. 2.6%, *p* = 0.29).^11^

In addition to the prospect of avoiding unnecessary cardiac testing for low-risk patients, women evaluated with the ASGES are also more likely to receive an accurate diagnosis. Comparative data from the PREDICT (Personalized Risk Evaluation and Diagnosis in the Coronary Tree) and COMPASS studies have shown that the ASGES outperforms Diamond–Forrester as well as Morise scores in the evaluation of current likelihood of obstructive CAD among symptomatic patients.

In the PREDICT study, receiver-operating characteristics (ROC) analysis for prediction of obstructive CAD showed a higher area under the curve (AUC) for the ASGES and Diamond–Forrester risk score combination than for the Diamond–Forrester risk score alone (AUC, 0.72 vs. 0.66; *p* = 0.003).^9^ In the COMPASS study, a similar ROC analysis yielded values of 0.79, 0.67, and 0.69 for the ASGES, Morise, and Diamond–Forrester scores, respectively (*p* < 0.001). Furthermore, in the COMPASS study, the ASGES (ROC 0.79) outperformed MPI (ROC 0.63) in ROC analyses for obstructive CAD discrimination.^10^ These findings emphasize the role of ASGES as a tool to aid in the diagnostic evaluation of women with suspected CAD.

Improving the efficiency of the diagnostic process for suspected obstructive CAD is of particular importance in women.^23^ Patients invest a significant amount of resources in the form of time and out-of-pocket expenses during an evaluation for CAD, and opportunity costs of these investments include time away from work and other productive or valued activities. Patients also frequently bear risks associated with exposure to radiation and contrast agents during their evaluation.^24, 25^ While the opportunity costs of evaluation are common to both men and women, the risks associated with radiation and contrast agents are heightened in women, due to radiological susceptibility of breast tissue and the risk of contrast-induced injury to the kidney and thyroid.^24–27^ From a societal perspective, the ASGES has been shown to be cost-effective and may reduce overall health care expenditures.^28,29, 30^

In this cohort, we also observed that many women with elevated scores (56%) were not referred for further cardiac evaluation. This finding may be attributable to multiple causes. For example, patients may have preferred to avoid subsequent testing, physicians may have recommended medical management with initiation or titration of preventive medications such as statins or beta blockers, or patients' symptoms may have resolved. A recent meta-analysis similarly demonstrated analogous variability in referral to cardiac catheterization after normal or abnormal cardiac stress test results.^31^ In addition, we also observed that 9% of women with low scores were referred for further cardiac evaluation. This finding may be attributable to multiple causes. For example, patients may have preferred to have subsequent testing, and/or physicians may have been concerned about the severity of the patient's symptoms despite the low score. Importantly, the test is not designed to be prescriptive in nature. Rather, we advise that clinicians use the ASGES—in the context of other clinical information—to determine the current likelihood of CAD and whether further cardiac testing is necessary.

Similar variability was demonstrated in a large registry of patients referred for MPI, positron emission tomography, or CCTA.^32^ Furthermore, the referral rates in low ASGES women we now report are higher than the referral rate reported from the combined cohort of women from the REGISTRY I and IMPACT-PCP studies, two other clinical utility studies on the ASGES.^14^ We are uncertain of the reasons for these differences, but this finding may be due to the fact that physicians in this PRESET Registry practiced in a larger number (21 vs. 8) of United States primary care practices and were less experienced with the use of the ASGES compared with physicians in the IMPACT-PCP or REGISTRY-1 studies.

Our study has several limitations. The absence of a control group in the PRESET Registry limits inferences about overall effectiveness compared with usual care, although our logistic regression analyses demonstrated a relationship between low ASGES and decreased cardiac referrals that is independent of several demographic and clinical characteristics. Nonetheless, these data from a real-world registry do not allow us to directly infer the incremental impact of ASGES on decision-making, relative to usual care. Second, because the focus of this study was clinical decision-making, we did not perform additional validation tests as part of the study protocol. Furthermore, we were underpowered to statistically assess MACE rates. A low MACE rate has been observed recently in other studies, such as the NIH-sponsored PROMISE Trial (NCT01174550), where the MACE rate (including procedural complications) among 10,003 patients referred for advanced cardiac testing was 3% at 2-year follow-up.^33^

Contemporary studies may require substantially larger study populations to robustly evaluate this outcome. However, the clinical validation studies PREDICT, COMPASS, and PROMISE substudy have previously reported robust test performance along with safety and long-term follow-up data. Of note, these clinical validation studies did not consider the contribution of microvascular disease as a cause of patients' symptoms, which may occur without obstructive CAD and is associated with worse outcomes among women compared to men.^34, 35^ Third, although baseline characteristics of patients in the PRESET Registry are comparable to other cohorts of patients with suspected CAD, the PRESET population may differ in other meaningful ways that may limit generalizability. Of note, the ASGES has been shown to have similar diagnostic characteristics among Caucasian and non-Caucasian populations. Specifically, in an analysis of the PREDICT and COMPASS trials, we performed a subanalysis that included 138 non-Caucasian and 1,364 Caucasian patients. This subanalysis demonstrated very similar ASGES performance between these two populations (AUCs = 0.72 vs. 0.70, respectively).^36^

Fourth, in this real-world setting, we observed that ∼4% of patients had contraindications for use of the ASGES test (3% autoimmune or inflammatory disease and 1% systemic inflammatory disease). In addition, the results of this study cannot be generalized to patients for whom the ASGES has not been validated, such as patients with diabetes. During the development of the ASGES, it was discovered that distinct genes are dysregulated in diabetic patients with obstructive CAD versus without nondiabetic patients with obstructive CAD.^15^ This finding may be due to differences in pathophysiology or may be related to diabetes-specific medications. A subsequent study also showed that genetic polymorphisms can have independent effects on CAD likelihood, specifically in diabetic patients.^37^

Finally, the results here represent a subgroup analysis of women from the larger PRESET Registry.^38^ Such subgroup analyses in women have been encouraged by several initiatives, including GoRedforWomen,^39^ Women's Heart Alliance,^40^ and CardioSmart (ACC) Women and Heart Disease.^41^ The work of Blum and Blum and McSweeney et al., among others, have provided the rationale for exploring sex differences in the evaluation of men and women with suspected obstructive CAD.^42, 43^ Furthermore, because of the challenges and risks associated with workup of women suspected of having obstructive CAD, the use of subgroup analyses in this setting may be justified: limiting subgroup analyses to those most likely to demonstrate greater benefit or harm, combined with formal testing and reporting of the subgroup results, may provide an opportunity to support safer, more cost-effective care.^44^

## Conclusions

A major challenge faced by primary care providers is the accurate and efficient evaluation of chest pain and related symptoms in women. In this large community-based registry, the ASGES demonstrated clinical utility among women evaluated for suspected obstructive CAD. Low ASGES was associated with lower rates of subsequent cardiac referral as well as low MACE rates and identified a population of women who were less likely to benefit from further cardiac evaluation.
